# Gasdermins in sepsis

**DOI:** 10.3389/fimmu.2023.1203687

**Published:** 2023-11-03

**Authors:** Wenhua Wang, Zhihui He

**Affiliations:** ^1^ Department of Intensive Care Unit, the Third Xiangya Hospital, Central South University, Changsha, Hunan, China; ^2^ Sepsis Translational Medicine Key Laboratory of Hunan Province, Central South University, Changsha, Hunan, China

**Keywords:** sepsis, gasdermin, regulation, cell death, therapy

## Abstract

Sepsis is a hyper-heterogeneous syndrome in which the systemic inflammatory response persists throughout the course of the disease and the inflammatory and immune responses are dynamically altered at different pathogenic stages. Gasdermins (GSDMs) proteins are pore-forming executors in the membrane, subsequently mediating the release of pro-inflammatory mediators and inflammatory cell death. With the increasing research on GSDMs proteins and sepsis, it is believed that GSDMs protein are one of the most promising therapeutic targets in sepsis in the future. A more comprehensive and in-depth understanding of the functions of GSDMs proteins in sepsis is important to alleviate the multi-organ dysfunction and reduce sepsis-induced mortality. In this review, we focus on the function of GSDMs proteins, the molecular mechanism of GSDMs involved in sepsis, and the regulatory mechanism of GSDMs-mediated signaling pathways, aiming to provide novel ideas and therapeutic strategies for the diagnosis and treatment of sepsis.

## 1 Introduction

Sepsis is a life-threatening organ dysfunction due to host response disorder caused by infection (1). Septic shock is a subtype of sepsis in which severe circulatory, cellular, and metabolic abnormalities occur, resulting in a significantly higher mortality than mono sepsis (2). With high morbidity and mortality (3), sepsis is the leading cause of death in critically ill patients (4). Globally, 48.9 million incident cases of sepsis were recorded in 2017, and 11 million sepsis-related deaths, accounting for 19.7% (18.2 ~ 21.4) of all deaths (5). Moreover, the morbidity of sepsis is increasing year by year with an annual growth rate of approximately 5.7% per year from 2007 to 2013 (6). This is mainly associated with improved diagnostic sensitivity, aging populations, the emergence of antibiotic-resistant strains, and increasing immunodeficient patients. In recent years, with the development of diagnosis and treatment and the standard of intensive care, the mortality of sepsis has decreased slightly (7). However, progress in clinical treatment strategies for sepsis remains slow, mainly due to the complexity of the disease (8). Sepsis can be caused by infections anywhere in the body. Pathogens contributing to sepsis include gram-positive bacteria, gram-negative bacteria, anaerobic, fungi, etc. The pathophysiological mechanisms of sepsis are complex, including imbalance of inflammatory response, endothelial dysfunction, coagulation disorders, imbalance of immune response, etc. (9). Clarifying the pathophysiological process of sepsis is essential for exploring therapeutic approaches.

With the better understanding of sepsis, the role of the dynamic changes of inflammatory reaction and immunosuppression in sepsis progression has become increasingly important, resulting in a highly heterogeneous state (10). In sepsis, pro-inflammatory and anti-inflammatory responses are activated simultaneously. In the early stage, under the stimulation of infection, the immune system is activated and releases pro-inflammatory cytokines and chemokines, presenting hyperinflammation (11). Among them, neutrophils are the earliest innate immune cells which migrate from blood to the infected sites (12). Through phagocytosis, degranulation, and neutrophil extracellular traps (NETs), neutrophils release reactive oxygen species (ROS), proteases, chemokines, and cytokines to kill pathogens and recruit other immune cells, maximizing the host’s immune response (12, 13). GSDMs-mediated cell death and inflammatory factor release aggravated the progress of pro-inflammation (14). If the performances of inhibiting the release of pro-inflammatory factors and regulating the body’s immune system are executed in time at this stage for restoring the balance between pro-inflammation and anti-inflammation, the host may recover to normal (15). Conversely, the interaction between various inflammatory factors and immune cells leads to “cytokine storm”, in which multiple systems such as reticuloendothelial system, complement system, and coagulation system are activated, causing tissue damage, and inducing cell death and even severe immune suppression (10, 11, 15). Furthermore, immature neutrophils suppressed proliferation and cell killing of lymphocytes aggravating the immunosuppression (16). Neutrophil dysfunction is positively correlated with the severity of organ dysfunction in sepsis (17). Specifically, neutrophils over-activated by complement components accumulate extensively and produce excessive pro-inflammatory cytokines, leading to tissue damage (18). NETs produced by neutrophils can damage endothelial cells, resulting in impaired microcirculatory blood flow and procoagulant alterations (12). Additionally, neutrophils promote intravascular coagulation through the NETs-platelet-thrombin axis, leading to microcirculatory dysfunction and tissue damage (19). The formation of NETs is dependent on proteases-activated GSDMs (20). Therefore, early intervention has become the focus of research to prevent sepsis from developing into uncontrollable stages. GSDMs as an important element in the progression of pro-inflammation in sepsis, it is crucial to elucidate its influence in sepsis. GSDMs proteins were first identified as being expressed in gastrointestinal and skin tissues and were named gasdermins (21, 22). Subsequently, studies have shown that GSDMs proteins are widely expressed in various tissues (23). The GSDMs family is a class of effectors that form pores in cell or organelle membranes during cell death and may contribute to multiple physiological and pathological processes such as epithelial cell restitution in inflammatory bowel disease, intestinal epithelium development (24, 25) and inflammation, carcinogenesis, dysregulation of immune response (26, 27). GSDMs proteins are activated by the stimulation of sepsis initiating factors and then cause inflammatory mediators release or inflammatory cell death, further aggravating sepsis progression.

In this review, we summarize the role of GSDMs in sepsis. Through the pathways in which GSDMs function to understand the contribution of GSDMs in the pathogenesis and progression of sepsis, it will be helpful to suggest potentially effective therapeutic strategies for shortening the course of sepsis and improving the prognosis and survival of septic patients.

## 2 GSDMs

**Figure 1:**
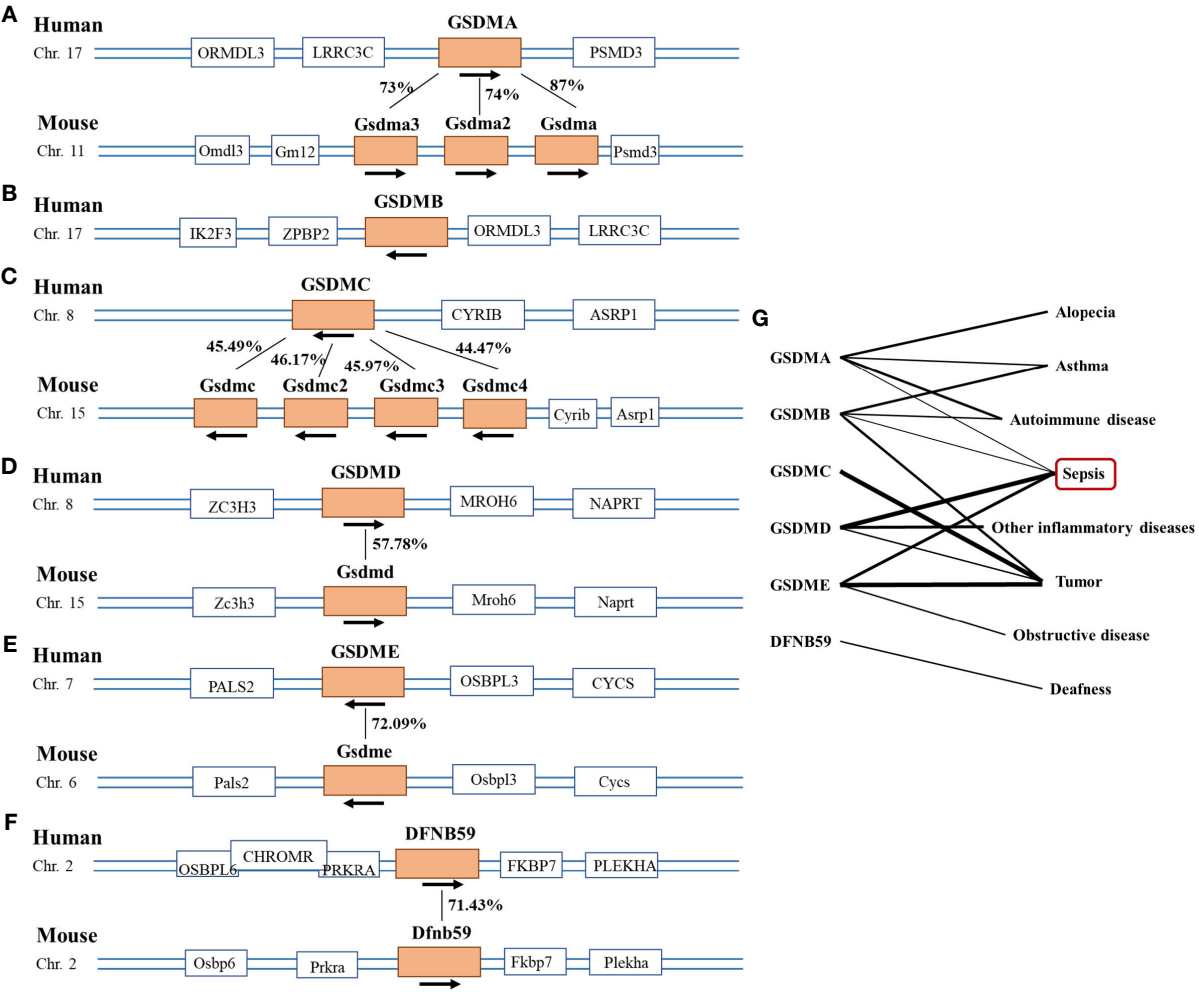
Picture of the Gasdermin family. **(A–F)** The location of the Gasdermin family on human and mouse chromosomes and the similarity of amino acid sequence between species. Human GSDMA has 87%, 74%, and 73% amino acid sequence similarity with mouse Gsdma, Gsdma2, and Gsdma3 respectively. And human GSDMC has 45.49%, 46.17%, 45.97% and 44.47% amino acid sequence similarity with mouse Gsdmc, Gsdmc2, Gsdmc3 and Gsdmc4 separately, which was 57.78% in GSDMD, 72.09% in GSDME and 71.43% in DFNB59. **(G)** Diseases caused by abnormal condition of the gasdemin family, the thickness of the lines represents the intensity of the correlation between the gene and the disease. The thicker the line, the stronger the correlation.

Evidence has shown that the GSDMs family is represented in fish, birds and mammals, with the most studied in humans and mice. Among the proteins encoded by the human genome, GSDMs protein have been classified into six groups: gsdermin A (GSDMA), gsdermin B (GSDMB), gsdermin C (GSDMC), gsdermin D (GSDMD), gsdermin E (GSDME), and Pejvakin (DFNB59). The GSDMs family is relatively conserved (28). Within the mouse genome, it has been divided into Gsdma1-3, Gsdmc1-4, Gsdmd, Gsdme, and Dfnb59 (29) (**Figure 1**). Except DFNB59, GSDMs are composed of two domains, an N-terminal with pore-forming function and a self-inhibiting C-terminal domain. The middle of the two domains is a linkage region with different lengths and sequences, which is the acting location of different enzymes to activate GSDMs proteins (28). DFNB59 contains only a truncated C-terminal domain, suggesting an entirely distinct activation mechanism from other members (30). Except DFNB59, GSDM proteins are activated for pore formation at the plasma membrane (14). Pyroptosis is the main way in which GSDMs play a role in sepsis (31). Recently, JOHNSON et al. revealed that bacterial genes produce proteins structurally similar to GSDMs proteins in mammals via X-ray crystallography. Both bacterial and human GSDMs are activated by a similar mechanism (32). This important finding revealed pyroptosis as an ancient programmed cell death common in bacteria and animals.

### 2.1 GSDMA

GSDMA is localized on chromosome 17 in humans and chromosome 8 in mice, which is highly expressed in gastrointestinal epithelium, epidermis and hair follicles. Correspondingly, there are three GSDMA alleles Gsdma 1-3 in mice. Gsdma 3 is highest in the skin, Gsdma 2 is highest in the stomach, and Gsdma is abundant in both the stomach and skin (33). GSDMA has been studied in gastric cancer, alopecia and susceptibility to inflammatory diseases (21, 34, 35). In addition, Streptococcus pyogenic exotoxin B (SpeB) could cleave and activate GSDMA (22). Streptococcus pyogenes, a potent toxigenic pathogen, can also cause toxic shock and sepsis through respiratory infection (22, 36). GSDMA has been poorly studied in sepsis, and GSDMA in sepsis needs further exploration.

### 2.2 GSDMB

GSDMB, also known as GSDML, is present only in the mammals, except in mice (37). The gene location and function of GSDMB and GSDMA are closely correlated, suggesting that both are produced by gene duplication (29). GSDMB has been detected as highly expressed in immune cells such as T cells (38, 39). GSDMB improved cell proliferation and migration, activated immune response, and regulated the processes of cell differentiation and cell death (40). GSDMB is associated with autoimmune disease and tumor progression (24, 41). GSDMB activation is a direct response to natural killer (NK) cells recognizing pathogen-infected cells (42). IpaH7.8 secreted by Shigella flexneri ubiquitinated and targeted GSDMB for 26S proteasome destruction to protect Shigella from the bacteriocidic activity of NK cells (43–45). GSDMB can also contribute to the development of sepsis by activating GSDMD (39). In addition, GSDMB was highly expressed in leukocytes of septic shock patients (39). These indicated the role of GSDMB in sepsis.

### 2.3 GSDMC

GSDMC is located on chromosome 8 in humans and chromosome 15 in mice, and is mainly expressed in esophagus, trachea, intestine and spleen tissues (46). GSDMC was first found with increased expression in metastatic melanoma and as a marker of melanoma progression (46). GSDMC is mainly related to tumor progression. GSDMC promoted proliferation and migration of tumor cell (35, 47). In macrophages, nuclear programmed death ligand 1 (PD-L1) converted TNF-α-induced apoptosis into pyroptosis by activating GSDMC (48). Gsdmc is the main effector of intestinal type 2 inflammation. After worm infection, GSDMC promoted the secretion of the “alarmin” cytokine interleukin-33 (IL-33) by intestinal epithelial cells to initiate type 2 responses for worm clearance and tolerance (49). Physiologically, Gsdmc N^6^-adenomethylation (m6A) conserved mitochondrial homeostasis and inhibited apoptotic pathways, critical for intestinal stem cell survival and maintenance of normal colonic epithelial regeneration (50). However, the role of GSDMC in sepsis has not been studied.

### 2.4 GSDMD

GSDMD is consistently localized on chromosomes with GSDMC in humans and mice, highly expressed in epithelial cells of the upper gastrointestinal and small intestinal mucosa, and immune cells such as macrophages, monocytes, neutrophils, and CD8^+^T cells (51). GSDMD cleavage was increased in activated CD8^+^ T cells, and GSDMD deficiency impaired the effector capacity of CD8^+^T cells (52). Among the gasdermins protein family, GSDMD was the first discovered protein to be cleaved by caspases and then generated an N-terminal domain with the ability to target the cell membrane, ultimately causing pyroptosis (53). Due to its wide distribution and the earliest discovery of mechanisms in pyroptosis, GSDMD is the most extensively studied protein in the pore-forming gasdermin protein family. GSDMD promoted the secretion of intestinal cupped cell mucin and the formation of mucus layer, contributing to homeostasis of the intestinal barrier (54).

GSDMD exerts essential effects on tumors and inflammatory diseases (55). The role of GSDMD in inflammatory diseases is clearly identified, mainly exerting pro-inflammatory effects through inflammatory cell death and the release of inflammatory mediators. Hypoxia/reoxygenation caused GSDMD-mediated cardiomyocyte pyroptosis and release of IL-18 (56). GSDMD contributed to the type II inflammatory response by promoting IL-33 release in pulmonary epithelial cells (57). Many studies have shown that GSDMD may promote the progression of sepsis by inducing pyroptosis and releasing inflammatory mediators (58–60). In neutrophils, GSDMD formed pores in organelle membranes, but not in cytomembrane. For example, GSDMD forming pores in azurophilic granules, elastase was released into the cytoplasm and mediated serine protease-dependent GSDMD cleavage. And forming pores in autophagosomes, GSDMD promoted to release IL-1β through autophagy-dependent pathways (61). In addition, Gsdmd-knockout (KO) mice protected against septic myocardial dysfunction, with a high survival rate (62). Downregulated GSDMD alleviated Candida albicans-associated sepsis (63). GSDMD is regarded as a novel ideal target for sepsis treatment.

### 2.5 GSDME

GSDME is located on chromosome 8 in humans and chromosome 15 in mice, and is clearly expressed in multiple tissues, including the brain, endometrium, placenta, and intestine (29). GSDME was first described in familial presbycusis, also known as deafness, autosomal dominant 5 (64). GSDME is closely associated with hear impairment, autoimmune diseases, and tumorigenesis (23, 30, 65).

GSDME plays an important role in inflammatory diseases. GSDME exerted protective effects in ultraviolet B-induced skin inflammation by inhibiting neutrophil over-recruitment and activation, thereby inhibiting cutaneous barrier damage (66). Cytokine storm is responsible for high mortality in sepsis patients. The synergism of TNF-α and IFN-β triggered human airway epithelial cells death by activating GSDME-mediated pathway (67). The H7N9 virus activated GSDME-mediated alveolar epithelial cell pyroptosis and inflammatory mediators release in mouse lungs, leading to cytokine storm and mortality in mice (68). GSDME activation is a critical and unique mechanism by which infection contributes to cytokine storm and lethality in sepsis. Thus, GSDME is a potential target for sepsis therapy.

### 2.6 DFNB59

DFNB59 is located on chromosome 2 in humans and chromosome 2 in mice, and is expressed in inner ear hair cells and other auditory system cells (69). DFNB59 has only been shown to be associated with hearing losses (70). Due to the distinctive structure, it is uncertain whether DFNB59 has the pore-forming capability. Currently, no studies have shown a correlation between DFNB59 and sepsis.

## 3 GSDMs in sepsis

### 3.1 GSDMs-mediated pathway

GSDMs proteins are activated by either caspases or granzymes, which is the basis for studies on GSDMs-mediated pathways. However, recent research has shown that GSDMs can also be identified and cleaved by other molecules except caspases and granzymes. Based on this classification, we clarified the following.

#### 3.1.1 GSDMs-mediated pathway including caspases or granzymes

Activated pattern recognition receptors (PRRs) on target cells combined with apoptosis-associated speck-like protein containing a caspase recruitment domain (ASC) to form inflammasome complexes containing nucleotide-binding oligomerization domain, leucine-rich repeat and pyrin domain-containing 1 (NLRP1), NLRP3, NLRC4 and absent in melanoma 2 inflammasome (71). The latter recruited and activated pro-caspase-1 (in the GSDMD-mediated canonical pathway) (72). Pro-caspase-4/-5/-11 (in the GSDMD-mediated noncanonical pathway) (73) and pro-caspase-3 (in the GSDME-mediated pathway) (74) could be activated independently of inflammasome. Caspases can also be activated in other ways. Yersinia triggered the interaction between receptor-interacting serine-threonine protein kinase 1 (RIPK1) and caspase-8, then activated caspase-8 (75). Caspase-11 enhanced the activation of caspase-8 to amplify inflammatory signals associated with tissue damage in sepsis (76). Activated caspases not only cleaved and activated the corresponding GSDMs proteins (35), but also cleaved prototypes of pro-inflammatory factors such as pro-IL-1/-18 (53). GSDME can be activated compensatively when the GSDMD signaling pathway is defective. In Gsdmd^-/-^ macrophages, NLRP3 inflammasome continuously induced caspase-8/-3 and GSDME cleavage and IL-1β maturation. Thus, when classical NLRP3-GSDMD signaling is blocked, the compensatory inflammatory pathway caspase-8/-3-GSDME is activated upon NLRP3 activation (77).

In addition to caspases, granzymes can also activate GSDMs proteins. In septic acute respiratory distress syndrome (ARDS), the expression of both GZMA and GZMB was upregulated (78). Specifically, GZMA secreted by NK cells and cytotoxic T lymphocytes (CTLs) directly activated GSDMB (38). GZMB secreted by CTLs or CAR-T cells could cleave GSDME directly at the site consistent with caspase-3 (79, 80). Also, GZMB cleaved caspase-3 to activate GSDME (81). In addition, GSDMs proteins can interact with each other. For example, GSDMB promotes the activity of caspase-4 by binding to the CRAD domain of caspase-4, thus cleaving GSDMD (39).

#### 3.1.2 GSDMs-mediated pathway except caspases and granzymes

GSDMs can also be cleaved and activated by some other molecules in sepsis, except well-known caspases or granzymes. A recent study showed that SpeB directly and specifically cleaved GSDMA in the junctional region after Gln246 (22). GSDMA functions both as a receptor to recognize exogenous pathogens and as an effector to form pores in the cell membrane and release inflammatory factors to trigger pyroptosis and inflammation. In addition, it has been shown that in lipopolysaccharide (LPS)-induced septic mice, Cathepsin G (Cat G), expressed on myeloid cells, can cleave human or murine GSDMD at Leu274, an upstream site of the caspase effector site Asp276, to form GSDMD-NT distinct from the cleavage of caspase-11 (82).

**Figure 2:**
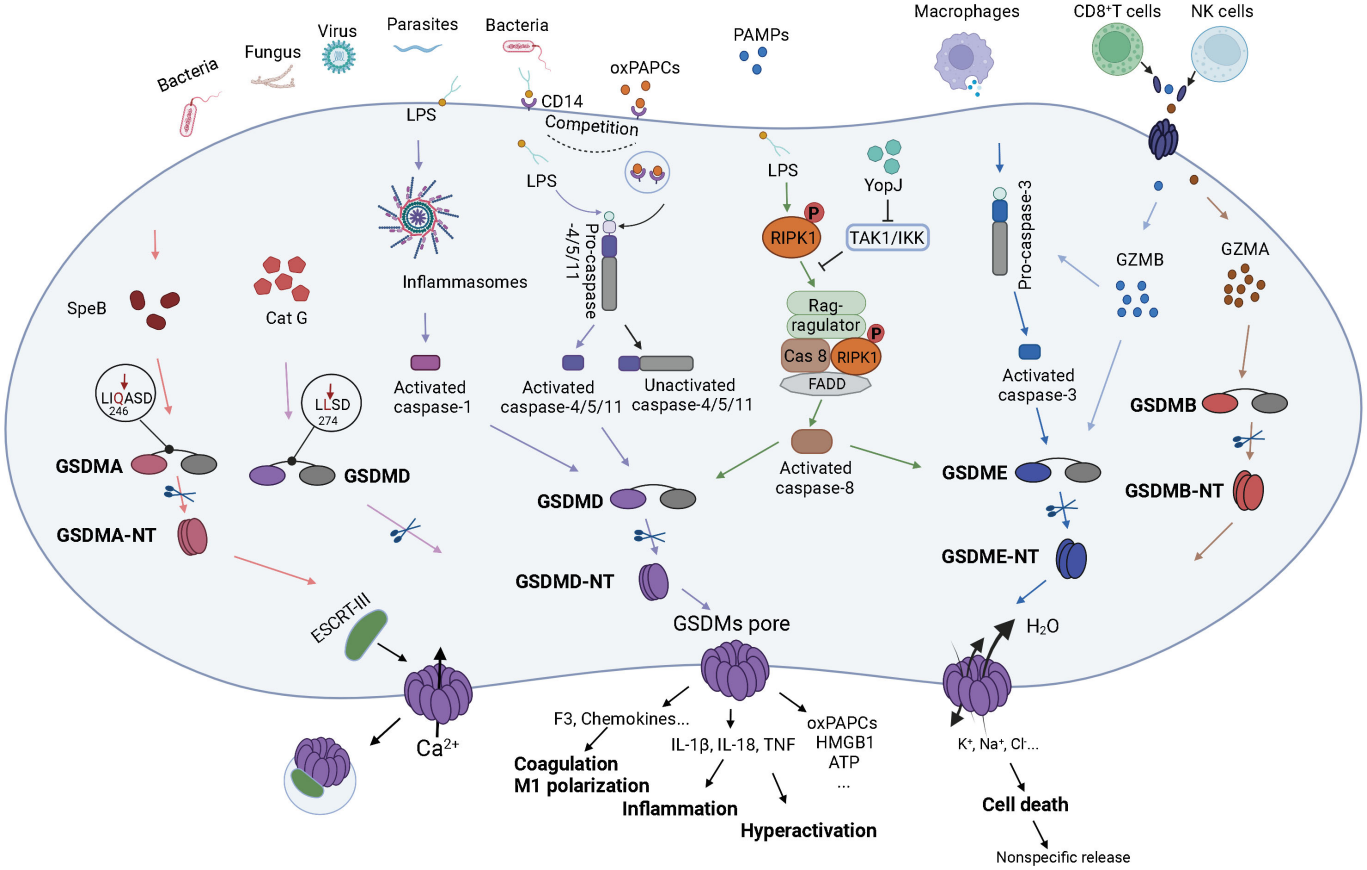
The molecular mechanism of GSDMs in sepsis. Bacterial, fungal, viral or parasitic severe infections or major traumas to body can cause sepsis, which in turn leads to systemic inflammatory response and imbalanced immune regulation. The above factors, as PAMPs or DAMPs, bind to PRRs on the surface of target cells and transmit signals intracellularly, ultimately activating GSDMs-related signaling pathways. SpeB, an exotoxin of Streptococcus pyogenes, can directly act the site after Gln264 in the GSDMA linkage region, which in turn cleaves GSDMA and forms pores in the cell membrane. Perforin and granzyme released by CD8^+^ T cells and NK cells can also impact the target cells. Specifically, GZMA can interact directly with GSDMB, causing GSDMB to be cleaved and pore formation on the cell membrane. Meanwhile, GZMB can not only activate pro-caspase-3 which in turn activates GSDME, but also can function directly on the cleavage site of GSDME to activate GSDME and thus pore forming. Notably, GSDMD plays the most important effect in sepsis. After the target cells receive the stimulatory signal, the inflammasome complex is assembled and thus activates caspase-1 which cleaves and activates GSDMD and pro-IL-1β. Upon entry into the cytoplasm, LPS exerts on CARD domain of pro-caspase-4/5/11, resulting in activation of caspase-4/5/11, and thus cleaves GSDMD. The oxPAPCs can then compete with LPS to bind the CD14 receptor on the cytosolic membrane and bind the catalytic domain of pro-caspase-4/5/11 to form inactive caspase-4/5/11. In addition, intracellular Cat G can also act directly the site after Leu274 on the GSDMD linkage domain to cleave GSDMD. The virulence of Yersinia, acetyltransferase YopJ-induced inhibition of TAK1 or IKK could recruit the RIPK1- FADD- caspase-8 complex to the Rag-ragulator platform to activate caspase-8 which leaded to macrophages pyroptosis by activating caspase-8/GSDMD or caspase-8/GSDME pathways. Activated GSDMD can also form pores in the cytosolic membrane. GSDMs pores can cause massive H_2_O molecules into the cell and the ion imbalance of the cell, which in turn leads to cell death and release of cell contents. GSDMs pores can also specifically release molecules, including inflammatory cytokines IL-1β, IL-18, TNF to promote inflammatory responses, coagulation factor F3 and chemokines contributing to the coagulation responses and M1 macrophage polarization, as well as active components such as oxPAPCs, HMGB1, ATP to activate other target cells and initiate the inflammatory cascade. In partial target cells, calcium enters the cells and triggers ESCRT-III to interact with GSDMs pores, causing some GSDMs pores to detach from the cell membrane and preventing cell death while continuously releasing inflammatory substances, which eventually result in over-activation.

GSDMs-mediated pathways are regulated by various molecules, such as miRNAs, oxidative stress, chemokines (83–86). Activated GSDMs proteins bound to the plasma membrane and formed pores (87), mediating cell death or inflammatory factors release, functional proteins, etc. (88–90) (**Figure 2**).

### 3.2 The activation of GSDMs-mediated pathway in sepsis

Sepsis is caused by a dysregulated inflammatory response to the presence of pathogenic microorganisms. PAMPs are highly conserved components of microorganisms’ surface and can be rapidly recognized by PRRs on the host cell surface, which in turn activate signaling pathways associated with GSDMs. PAMPs in sepsis mainly comprise surface membrane components of microorganisms (e.g., lipopolysaccharide, LPS) and microbial nucleic acids (e.g., DNA and RNA) (91). LPS, the main component of the cell wall of gram-negative bacteria, is the most important PAMP and is inextricably related to sepsis.

Clinically, the severity of sepsis has been shown to be correlated with danger-associated molecular patterns (DAMPs) (92–94). DAMPs could be released extensively via GSDMs-mediated pore-forming membrane or cell lysis, and subsequently could be recognized by PRRs and activate the GSDMs-mediated pathway (95). DAMPs are divided into many types, and sepsis-related ones include high mobility group box 1 (HMGB1), histones, ATP, uric acid, DNA, mitochondrial DAMPs, and IL-33 (96, 97). HMGB1 is the first to be identified and also the most intensively studied in sepsis by binding to receptors for advanced glycation end-products (RAGE) and toll-like receptor 2 (TLR2)/TLR4 (91). LPS induced HMGB1 release from hepatocytes into exosomes through the coordinated activities of TLR4 and caspase-11/GSDMD signaling (98). In turn, HMGB1 released from hepatocytes interacted with LPS and is internalized into the lysosomes of macrophages and endothelial cells by binding the RAGE, mediating the caspase-11/GSDMD signaling pathway (99).

### 3.3 The regulation of GSDMs-mediated pathway in sepsis

The GSDMs-mediated pathway plays a crucial role in sepsis. The main elements of the GSDMs signaling pathway have been well studied (88, 100). However, molecular mechanisms regulating this pathway have not been systematically summarized. In this review, regulation of the GSDMs-mediated pathway was synthesized from multiple perspectives and listed in **Table 1**.

**Table 1:** The regulation of GSDMs-mediated pathway in sepsis.

Gasdermin	Regulator	Model	Source of regulator	Effective cell	Effector	Regulation Approach	Ref (PMID)
Epigenic regulation							
GSDMD	miR-30d-5p	Sepsis-induced ALI mice	Neutrophil	Macrophages	M1 macrophage polarization and pyroptosis	Exosomal miR-30d-5p upregulated NLRP3 inflammasome expression through NF-κB signaling pathway	34641966
GSDMD	miR-21	LPS-induced septic shock	LPS-induced macrophages	BMDMs	Pyroptosis	miR-21 promoted NLRP3-induced pyroptosis in septic shock by activating NF-κB pathway via A20.	31189875
GSDMD	circ-HIPK3/ miR-124-3p	Sepsis-induced AKI mice	Serum from patients or mice	TMCK-1 cells	Pyroptosis	circHIPK3 upregulated KLF6 expression by competitively binding to miR-124-3p, thereby promoting the binding of KLF6 and NLRP3, activating NLRP3/caspase-1 pathway	35576907
GSDMD	miR-223-3p	Sepsis-induced AKI mice	Renal tissue	TMCK-1 cells	Pyroptosis	KLF6 inhibited miR-223-3p via binding to the miR-223-3p promoter and promoted NLRP3, and activated the NLRP3/Caspase-1 pathway	34710881
GSDMD	miR-93-5p	Sepsis-induced AKI mice	Macrophages	TMCK-1 cells	Pyroptosis	Exosomal miR-93-5p activated NLRP3 through direct regulation of TXNIP to activated the NLRP3/Caspase-1 pathway	33745232
GSDMD	miR-30c-5p	Sepsis-induced AKI mice	Renal tissue	HK-2 cells	Inhibited pyroptosis	miR-30c-5p repressed the expression of TXNIP, which inhibited NLRP3, ASC, and caspase-1 expression	32892306
GSDMD	lncR-XIST/ miR-150-5p	Sepsis-induced MI rat	Myocardial tissue	H9C2 cells	Pyroptosis and apoptosis	lncRNA XIST/miR-150-5p/c-Fos axis regulated the promoter of TXNIP to activated the NLRP3/Caspase-1 pathway	34045679
GSDMD	miR-34a	Sepsis mice	Lung tissue	**—**	Pyroptosis	miR-34a activated caspase-1 and GSDMD through upregulation of ASC proteins in the inflammasome complex	32903604
GSDMD	circ-Katnal1/ miR-31-5p	Sepsis-induced liver injury mice	Liver tissues	Raw264.7 cell	Pyroptosis	circ-Katnal1 promoted the expression of GSDMD by impacting miR-31-5p to induce pyroptosis and liver injury	35979014
GSDMD	lncR MEG3/ miR-18a-3p	Sepsis-induced AKI mice	Renal tissue	TECs	Pyroptosis	lncR MEG3 promoted renal tubular epithelial pyroptosis by regulating the miR-18a-3p/GSDMD pathway in LPS-induced AKI	34012408
GSDMD	YTHDF1	CLP-induced mice	LPS-ATP induced RAW264.7 cells	RAW264.7 cells	Inhibited pyroptosis	YTHDF1 alleviated sepsis by upregulating the transcription of WWP1 to induce NLRP3 ubiquitination and inhibit caspase-1-dependent pyroptosis.	35508474
GSDMD/ GSDME	HDAC11	TNF-α induced HUVECs	HUVECs	HUVECs	Pyroptosis	HDAC11 might promote both NLRP3/caspase-1/GSDMD and caspase-3/GSDME pathways leading to pyroptosis via regulation of ERG acetylation in HUVECs	35279683
GSDME	YopJ	**—**	Myeloid cells	Neutrophil	Neutrophil pyroptosis to defence Yersinia	YopJ activated GSDME via RIPK1/caspase-8 pathway, which in turn resulted in myeloid cell pyroptosis to resist Yersinia infection	34260403
GSDMD/ GSDME	YopJ	**—**	Extrinsic Yersinia	murine macrophages	Pyroptosis	YopJ inhibited TAK1 or IKK and then activated caspase-8, resulting in cleavage of both GSDMD and GSDME in murine macrophages	30361383 30381458
GSDMD	KAT2B and KAT3B /H3K27ac	**—**	A. baumannii induced-BMDMs	BMDMs	Pyroptosis	A. baumannii increased binding of H3K27ac and the promoter of caspase-11 and the GSDMD via lysine acetyltransferase KAT2B and KAT3B, upregulating their expression and ultimately increasing caspase-11/GSDMD-mediated pyroptosis	29352265
GSDMD	zDHHC5/9	LPS-Nig induced THP-1 cells	THP-1 cells	THP-1 cells	GSDMD-NT membrane transposition	GSDMD palmitoylation at Cys191/Cys192 (human/mouse) caused GSDMD-NT membrane transposition and pore formation.	36945424 36865189
Oxidative stress							
GSDMD	mtROS	LPS-Nig induced J774A.1 cells	J774A.1 cells	J774A.1 cells	Pyroptosis	Mitochondrial ROS promote macrophage pyroptosis by inducing GSDMD oxidation	30860577
GSDMD	PDE4B	LPS-induced septic ALI	BMDMs in septic mice	BMDMs	Pyroptosis	PDE4B promoted pyroptosis in LPS stimulated lung injury model and macrophages by regulating ROS/Nrf2/NLRP3 activation	34644617
GSDMD	STING	Septic mice	THP-1 and BMDMs	THP-1 and BMDMs	Release of blood coagulation initiator F3	The STING-dependent increase in cytosolic calcium drives GSDMD cleavage and activation, which triggers the release of F3	32142632
GSDMD	ox-mtDNA	Septic mice	Pyroptotic platelet	Platelet from human and mice	Pyroptosis and excessive release of pro-inflammatory cytokines	Pyroptotic platelet-derived ox-mtDNA potentially promoted to release S100A8/A9, which activated the caspase-1/GSDMD pathway, inducing pyroptosis of platelets and eventually resulting in excessive release of pro-inflammatory cytokines	35967457
Other regulation							
GSDMD	IRF2	LPS-induced iMACs	iMACs and BMDMs	iMACs and BMDMs	Pyroptosis	IRF2 bound directly to GSDMD promoter to directly drive GSDMD transcription for the execution of pyroptosis.	31113851
GSDMD	IRF2	sepsis-induced AKI mice	Patients' serum and HK-2 cells	HK-2 cells	Pyroptosis	IRF2 promoted LPS-treated HK-2 cell pyroptosis by increasing the expression of caspase-4 and GSDMD, instead of affecting caspase-1, NLRP3, and ASC.	34992348
GSDME	IRF1	TNF-α induced mice	Intestinal epithelial cells	Intestinal epithelial cells	Pyroptosis	IRF1 was required for caspase-3 expression in IECs by binding to IRF1-binding sites in the caspase-3 promoter, in turn inducing pyroptosis by activating the GSDME pathway	34309645
GSDMD	Bcl2	LPS-Nig induced THP-1 cells	THP-1 cells	THP-1 cells	Pyroptosis	Bcl-2 could bind directly to GSDMD to yield fragments without pore-forming ability and suppressed NLRP1 oligomerization to inhibit cell pyroptosis.	31839993
GSDMD	cAMP	LPS-Poly(I:C) induced mice	LPS or E. coli-induced BMDMs	BMDMs and THP1 cells	Inhibited pyroptosis	cAMP metabolism controlled the activation of caspase-11 inflammasome and pyroptosis in sepsis.	31131320
GSDMD	Itaconate	LPS-induced mice	LPS-induced macrophages	LPS+ATP-induced BMDMs	Inhibited pyroptosis	Itaconate prevented full activation of caspase-1 and GSDMD in LPS-induced macrophages	33691097
GSDMD	MAC	LPS-induced THP-1 cells and human MDMs	PAMP and DAMP induced in tissues	THP1 cells and human MDMs	Pyroptosis	MAC triggered self-internalization promoting ASC oligomerization and NLRP3 inflammasome assembly, which mediated macrophage pyroptosis via caspase-1/GSDMD.	34650553
GSDMD/ GSDME	LPS	RAW264.7 cultured with high glucose	Extrinsic pathogenic bacteria	RAW264.7	GSDMD mediated pyroptosis	LPS increased GSDMD expression partly while decreasing GSDME expression to induce pyroptosis in a GSDMD-dependent manner in high-glucose environments	35006493
GSDMD	MIF	CLP-induced septic mice	Kidney in septic mice	LPS-induced HK2 cells	Pyroptosis	Up-regulated MIF in septic mice aggravated kidney damage by increasing NLRP3/ GSDMD mediated cell pyroptosis.	35165294
GSDMD/ GSDME	IL-6	S. pneumoniae-treated mice	Endogenous cytokines during infection	RAW264.7 cells and alveolar macrophages	Inhibited pyroptosis	Up-regulated IL-6 caused by bacterial infection inhibited GSDMD/GSDME-mediated pyroptosis by suppressing caspase-1/3 activation and thus play partly protective role in bacterial infection.	35297653

NLRP3, NOD-like receptor 3; TMCK-1, Transformed C3H Mouse Kidney-1; HK-2, Human Kidney-2; H9C2, rat myocardial cells; MI, myocardial injury; AP, acute pancreatitis; TECs, Renal Tubular Epithelial Cells; HUVECs, human umbilical vein endothelial cells; BMDMs, Bone Marrow-derived Macrophages; Nig, Nigericin; J774A.1 cell, Mouse monocyte macrophages; THP-1 cells, human myeloid leukemia mononuclear cells; iMACs, immortalized macrophage line; U937; iPSC, induced pluripotent stem cell;HK2 cells, human kidney-2 cells.

**—** means the study do not involve this project.

#### 3.3.1 Epigenetic regulation in GSDMs-mediated pathway

Among the many regulatory mechanisms, epigenetic regulation plays an important role. Epigenetic mechanisms are a major way of regulating gene expression, and their core is multiple covalent modifications of nucleic acids and histones reversibly and dynamically, mainly including DNA methylation, non-coding RNA (ncRNA) regulation and histone post-translational modifications (101, 102). Among them, ncRNA regulation and histone post-translational modifications play important roles in GSDMs-mediated signaling pathways in sepsis.

In the human genome, only about 2% of RNAs can be translated into proteins (103). The remaining RNAs are known as ncRNA, particularly long non-coding RNA (lncRNA), microRNA (miRNA) and circular RNA (circRNA) have been identified as critical for regulating the GSDMs pathway in sepsis (104). ncRNA may affect multiple components of the GSDMs pathway in sepsis. First, ncRNA could impact the inflammasome complex. In septic acute lung injury (ALI), the exosomal miR-30d-5p of neutrophils partially activated NF-κB pathway in macrophages and upregulated the expression of NLRP3 to mediate M1 macrophage polarization and pyroptosis (105). miR-21 promoted NLRP3 inflammasome activation to mediate pyroptosis and septic shock by activating NF-κB pathway (106). Circ-HIPK3 upregulated the expression of Krüppel-like factor 6 (KLF6) by competitively binding miR-124-3p. KLF6 not only directly upregulated NLPR3 (107) but also promoted NLRP3 by inhibiting miR-223-3p promoter activity (108) to activate NLRP3/Caspase-1/GSDMD pathway and accentuate septic acute kidney injury (AKI). Exosomal miR-93-5p activated NLRP3 by regulating thioredoxin-interacting protein (TXNIP), contributing to renal epithelial cell pyroptosis in septic mice (109). Downregulated miR-30c-5p triggered NLRP3/caspase-1/GSDMD pathway by promoting expression of TXNIP in septic AKI (110). Meanwhile, the lncRNA XIST/miR-150-5p/c-Fos axis exacerbated septic myocardial injury by regulating the promoter of TXNIP (111). These findings suggest that TXNIP may be a potential therapeutic target in sepsis. miR-34a upregulated expression of ASC protein to activate caspase-1/GSDMD and exacerbate septic ALI (112). In addition, ncRNA may affect the expression of GSDMs. Circ-Katnal1 promoted the expression of GSDMD by influencing miR-31-5p to enhance pyroptosis in septic liver injury (84). LncRNA MEG3 promoted renal tubular epithelial pyroptosis by regulating miR-18a-3p/GSDMD pathway in LPS-induced AKI (113). The studies showed multiple regulation of GSDMs-mediated pathway by ncRNA, suggesting that ncRNA is promising as biomarkers in sepsis.

Post-translational modifications of histones in GSDMs are essential in sepsis. YTH N^6^-Methyladenosine RNA Binding Protein 1 (YTHDF1), m6A reader protein, induced NLRP3 ubiquitination and inhibited caspase-1-dependent pyroptosis to alleviate sepsis (114). Histone deacetylase 11 (HDAC11) plays an important role in sepsis. In TNF-α-treated human umbilical vein endothelial cells, upregulated HDAC11 decreased the acetylation of ETS-related gene (ERG) to promote NLRP3/caspase-1/GSDMD and caspase-3/GSDME pathway (115). In myeloid cells, acetyltransferase Yersinia outer protein J (YopJ) activated GSDME but not GSDMD by promoting RIPK1/caspase-8 pathway to induce myeloid cell pyroptosis and protect host from Yersinia infection (116). Yet, in another study, YopJ-induced inhibition of TGF-α-activated kinase 1 (TAK1) or IκB kinase (IKK) could activate GSDMD and GSDME by activating caspase-8 to induce macrophages pyroptosis and exacerbate the inflammatory response (75, 117, 118). In infected macrophages, lysine acetyltransferase KAT2B and KAT3B increased the binding of the acetylation of histone H3 Lysine 27 (H3K27ac) to the promoters of caspase-11 and GSDMD to upregulate the expression of caspase-11 and GSDMD and increase caspase-11/GSDMD-mediated pyroptosis (119). Protein s-palmitoylation is a type of lipidation modification, which lipidates cysteine (Cys) residues via thioester bonds to target proteins towards organelles and plasma membranes (120). GSDMD palmitoylation at Cys191/Cys192 (human/mouse) caused GSDMD-NT membrane transposition, and only palmitoylated GSDMD-NT was enabled for membrane transposition and pore formation. GSDMD palmitoylation was modified by palmitoyl acyltransferases zinc finger DHHC domain 5 (zDHHC5) and zDHHC9, promoted by LPS-induced ROS (121, 122). At present, post-translational modifications on histones of GSDMs are still poorly studied in sepsis, and many regulatory mechanisms have yet to be discovered to enrich the role of GSDMs in sepsis.

#### 3.3.2 The regulation of oxidative stress in GSDMs-mediated pathway

Oxidative stress can impair multiple systems in the body by affecting the GSDMs pathway, which is a significant contributor to multiple organ dysfunction in sepsis (123). In macrophages, mitochondrial ROS (mtROS) oxidized the four amino acid residues of GSDMD to promote cleavage of GSDMD (58). Phosphodiesterase 4B (PDE4B) promoted activation of ROS/nuclear factor erythroid2-related factor 2 (Nrf2)/NLRP3 to induce inflammasome activation and pyroptosis in LPS-induced ALI (59). In sepsis, DNA damage may contribute to the activation of Transmembrane protein 173 (TMEM173, also known as STING), an endoplasmic reticulum (ER) stress-associated immune adaptor protein. TMEM173 promoted calcium release from macrophages and monocytes ER, leading to activation of caspase-1/-11/-8 in a bacterial type-dependent manner (e.g., activated caspase-1/-11 in E. coli infection and activated caspase-8 in S. pneumoniae infection). Platelets play an important role in the pathogenesis of sepsis, and the expression of GSDMD in platelets is significantly upregulated in septic patients. Mechanistically, pyroptotic platelet-derived oxidized mitochondrial DNA (ox-mtDNA) promoted the release of S100A8/A9 to activate the caspase-1/GSDMD pathway, inducing pyroptosis of platelets and ultimately forming a positive feedback loop that leads to excessive release of pro-inflammatory cytokines (124). Another *in vivo* murine experiment demonstrated that PD-L1 knockdown inhibited the expression of caspase-3 to reduce GSMDE-induced IL-1β release and suppressed the activation of integrin αIIbβ3, contributing to alleviate platelet activation in sepsis (125).

#### 3.3.3 Other regulation in GSDMs-mediated pathway

The interferon regulatory factor (IRF) family also had significant effects on the signaling pathway of GSDMs in sepsis. IRF2 can directly drive GSDMD transcription by engaging the promoter of GSDMD to induce pyroptosis in sepsis (126). IRF2-KO septic mice exhibited enhanced survival rates and reduced pathological manifestations (127). IRF1 enhanced caspase-3 expression by binding to its promoter, thereby inducing pyroptosis by activating the GSDME pathway (128). As regulators of the mitochondrial apoptotic pathway, members of the B-cell lymphoma-2 (Bcl-2) family influence the GSDMs pathway in sepsis. In LPS-treated macrophages, Bcl-2 recognized the BH3 domain on GSDMD-NT, promoting caspase directed cleavage of GSDMD at D87, which lacked pore-forming capability, instead of at D275. Furthermore, Bcl-2 impeded NLRP1 oligomerization by reducing the binding of ATP to NLRP, thereby inhibiting the activation of NLRP1 inflammasome (129). Immunometabolism represents a promising therapeutic target in sepsis. Activation of caspase-11 inflammasome and pyroptosis in sepsis was regulated by cAMP metabolism (130). In addition, itaconate, a unique regulatory metabolite, prevented full activation of caspase-1 and GSDMD in LPS-induced macrophages (131).

In sepsis, activation of the complement system results in the generation of membrane attack complex (MAC). MAC promoted ASC oligomerization and NLRP3 inflammasome assembly to activate caspase-1/GSDMD and induce macrophage pyroptosis (132). In addition to acting as PAMPs to initiate the GSDMs signaling pathway, LPS may also affect the expression of GSDMs proteins (133). LPS increased GSDMD expression while decreasing GSDME expression through glycolysis in RAW264.7 cells. And this transcriptional regulation suggested that LPS may contribute to pyroptosis in a GSDMD-dependent manner in high-glucose environments (134). However, the exact mechanism remains unclear and needs to be further explored. Upregulated macrophage migration inhibitory factor (MIF) increased NLRP3 inflammasome mediated cell pyroptosis and aggravated kidney damage by promoting phosphorylation of p65 in septic mice (135). Upregulated IL-6 caused by bacterial infection inhibited caspase-1/-3 activation to suppress GSDMD-/GSDME-mediated pyroptosis and played a partially protective role (136). Notably, the NF-κB signaling pathway exhibits a dual role in sepsis. On the one hand, activated NF-κB pathway reduced mortality in cecal ligation puncture (CLP)-induced septic mice by promoting M1 macrophage polarization and enhancing bacterial phagocytosis of macrophages (137). On the other hand, NF-κB signaling was required for the GSDMD-mediated pyroptosis in LPS-induced adipocytes (138). The role of NF-κB signaling pathway in sepsis remains to be investigated more deeply.

### 3.4 The effector of GSDMs-mediated pathway in sepsis

After GSDMs are cleaved, GSDMs-NT transfer to the membrane and perform pore-forming functions. This is mainly through selective binding of GSDMs-NT to phosphatidylinositol phosphate, phosphatidylserine and phosphatidic acid of the inner cell membrane, or to cardiolipins of damaged outer mitochondrial and bacterial membranes (28, 87), forming a 10-14 nm pore containing 24-34 symmetrical subunits (139). Cytoplasmic calcium signaling is a prerequisite for of GSDMD-NT translocation to the plasma membrane (133, 140). Mg^2+^ blocked Ca^2+^ influx by inhibiting ATP-gated Ca^2+^ channel P2X7 (140). And phospholipase C increased cytoplasmic calcium to affect GSDMD pore formation (133). Pore-forming cells have two outcomes and exert different effects *in vivo*.

#### 3.4.1 GSDMs-induced cell death

In sepsis, the most common effect of GSDMs proteins is to induce pyroptosis, which leads to cellular rupture and non-specific release of cellular components, such as HMGB1 and pro-inflammatory cytokines such as IL-1, IL-18 and TNF-α, ultimately triggering inflammatory responses (141). Specifically, after pore formation, the cell membrane loses integrity, the cell ion gradient is depolarized, and the osmotic pressure on both sides of the membrane is imbalanced, which ultimately causes rupture of the cell membrane and release of cell components (100, 142). This result is GSDMs-mediated final effect in most conditions and has been extensively studied.

#### 3.4.2 GSDMs-mediated cellular hyperactivation

In addition to pyroptosis, GSDMs can also cause cells to become hyperactivated (143). It has been shown that in macrophages, dendritic cells and neutrophils, GSDMD pores may induce cell hyperactivation and secrete cytokines (143, 144). This is mainly associated with endosomal sorting complexes required for transport (ESCRT)-dependent repair of plasma membrane pores (145, 146) and stimulation of oxidized phospholipids (oxPAPCs) (147). After GSDMD-NT pore formation at the plasma membrane, ESCRT, specifically ESCRT-III protein, was specifically recruited to the inner layer of the plasma membrane (148). With calcium influx as a signal, ESCRT repaired the damaged membrane region in a punctate pattern, which was verified by calcium chelation increasing macrophage pyroptosis (146). ESCRTs removed GSDMs pores from the plasma membrane by forming ectosomes. After a portion of the GSDMs pores were removed, ESCRT-III began to separate from the pore-forming regions on the plasma membrane (148). Consequently, ESCRT-mediated membrane repair not only maintained cellular activity, but also preserved part of the GSDMs pores to secrete pro-inflammatory cytokines (143, 144).

In addition, GSDMs-mediated cellular hyperactivation is also associated with oxPAPCs. oxPAPCs are components of oxidized low-density lipoprotein that are present in apoptotic cells and are LPS mimic (149). Similar to LPS, oxPAPCs could bind to CD14 on membranes. CD14 transmitted LPS and oxPAPCs to TLR4 receptors on the cell membrane, and then transported LPS and TLR4 into cytosol (150), resulting in CD14 endocytosis and depletion (147, 151). Thus, oxPAPCs competed for CD14 binding with LPS. oxPAPCs acted on the catalytic domain of caspase-11 and generated the cleavage fragment with very low activation activity different from that of LPS. *In vitro* experiments showed that oxPAPCs blocked LPS-induced caspase-11 cleavage in a dose-dependent manner (152). In addition to the plasma membrane, the GSDMD-NT dynamically binds to abundant intracellular organelle membranes. In neutrophils, GSDMD-NT is transported to azurophilic granules and autophagosomes, releasing IL-1β through autophagy-dependent pathways (61), which provide the rationale for neutrophil hyperactivation in sepsis. Therefore, innate immune cells can achieve different activation states to better defend against infection in sepsis.

#### 3.4.3 Active mediator release via GSDMs pores

In hyperactivated cells, the GSDMs pore preferentially releases mature IL-1β rather than pro-IL-1β. Mature and pro-IL-1β are both significantly smaller than the pore, of size 4.5 nm, suggesting that other factors affect transportation, not size. Pro-IL-1β and GSDMD pore are negatively charged and mutually repulsive. Since caspase-1 has cleaved the acidic domain of pro-IL-1β, IL-1β is positively charged, together with the electrostatic interaction of the pore, mature IL-1β can pass through more easily (139, 153).

In addition to the well-studied pro-inflammatory cytokines IL-1β and IL-18, other active components can be released from GSDMs pores, most commonly DAMPs. DAMPs could be recognized by multiple cells. Activated by DAMPs, innate immune cells can release pro-inflammatory mediators, leading to recruitment of inflammatory cells. DAMP induced the death of non-immune cells, disrupting tissue structure and homeostasis (154, 155) (**Table 2**).

**Table 2:** Active mediator released by GSDMs pores in sepsis.

Active substances	Characters	Roles in sepsis	Ref (PMID)
IL-1β, IL-18	Pro-inflammatory cytokines, the member of IL-1 family	Aggravating MODS by affecting multiple systems of the body	34472725 33187725
DAMPs	Endogenous molecules released by damaged or dead cells	Promoting the release of pro-inflammatory cellular mediators and exacerbating tissue damage	31736963 35418465
F3	Initiator of the extrinsic coagulation pathway	Causing coagulation disorder and DIC	32142632 31076358 14576054
Ferritin	Intracellular iron-storage protein	Depositing in tissues and causing tissues damage	30731209
K^+^	Inducing the generation of IFN-β	Protecting tissues from inflammatory mediators	30170814
mtDNA	—	Suppressing endothelial proliferation and impeding endothelial regeneration	32164878

MODS, multiple organ dysfunction.

**—** means the study do not involve this project.

In addition, the GSDMs pores can release other substances (**Table 2**). In sepsis, macrophages or monocytes released coagulation factor III (F3) via the GSDMD pore (60). F3 is an initiator of the extrinsic coagulation pathway and plays an important role in the pathophysiology of disseminated intravascular coagulation (DIC). Blocking F3 prevented endotoxemia and DIC in mice (156, 157). In LPS-induced sepsis, caspase-11/GSDMD pathway activation promoted the release of ferritin from macrophages and increased serum ferritin concentrations (89). Septic patients have a significantly poor prognosis associated with serum ferritin, which may act as a biomarker in sepsis (158). Because serum ferritin is difficult to excrete from the body, increased serum ferritin is deposited in tissues, causing tissue injury and increasing the severity of sepsis. Due to the body’s self-protective mechanisms, GSDMs pores also release some protective signals, such as potassium ion efflux before cell death (159). Although caspase-11/GSDMD activation-mediated potassium efflux is mildly responsible for triggering NLRP3 inflammasome formation and IL-1β activation (160). More importantly, cytoplasmic K^+^ enhanced the binding of dsDNA to the DNA sensor cyclic GMP-AMP synthase (cGAS) and induced the production of type I interferon (IFN-β) by STING pathway, ultimately causing tissue injury in sepsis. K^+^ efflux inhibited the generation of IFN-β and alleviated inflammatory response (161). GSDMD pores on mitochondria caused mtDNA release into the cytoplasm, and inhibited cell proliferation via cGAS/STING pathway (142).

#### 3.4.4 The shift of the way of cell death mediated by GSDMs

GSDMs proteins could also mediate the switch between different ways of cell death. Apoptosis and pyroptosis coexist in sepsis (162, 163). Since caspase-3/-8 are both the core of apoptosis and pyroptosis, GSDMs-mediated conversion of cell death between pyroptosis and apoptosis is the most common. Caspase/granzyme-induced apoptosis can be converted to pyroptosis due to the high expression of GSDMs. When the expression of GSDMs is too poor to induce pyroptosis, activated GSDMs may induce apoptosis. AKI-induced autophagy can activate GSDME by activating the caspase-8/-9/-3 apoptotic pathway to trigger pyroptosis. By knocking down autophagy-specific genes atg5 and fip200 or using apoptosis inhibitors, GSDME cleavage was inhibited (164). This research also linked autophagy, apoptosis and pyroptosis, demonstrating the importance of GSDMs proteins in cell death. GSDMs proteins could also promote other ways of cell death. NETosis is a novel type of cell death that differs from apoptosis and necrosis, and the death of neutrophils that occur during NETs formation, including “vital NETosis” and “suicidal NETosis “ (165). The former involves plasma membrane rupture and neutrophil lysis, whereas the latter refers to NETs release from neutrophils while maintaining intact plasma membranes (166). GSDMD is an essential regulator of NETosis (20). Inhibiting GSDMD reduced the production of NETs (167). Studies showed that depletion of GSDMD only on neutrophils worsened the severity of sepsis in mice, while systemic depletion of GSDMD alleviated the severity of sepsis (167, 168), highlighting the crucial role of NETosis in sepsis. In addition, when inflammasomes are activated in macrophages, increased mtROS promoted migration of GSDMDs to the mitochondrial membrane. Mitochondria released mtROS via GSDMD pores, which promoted RIPK1/RIPK3/mixed lineage kinase domain-like-dependent necrosis. The study demonstrated that mitochondrial dysfunction can affect immune outcomes through cell death modality switching (169). Overall, as executors of multiple cell death pathways, GSDMs perform the critical function in sepsis.

#### 3.4.5 Other effects of GSDMs-mediated pathway

Candida albicans promoted itself to escape from macrophages through GSDMD-mediated pyroptosis, which in turn released candidalysin and caused fungal sepsis (63). The GSDMs pathway may also contribute to the polarization of M1 macrophages (105). The deeper mechanisms by which GSDMs affect macrophage polarization still need further study.

## 4 The therapy targeting GSDMs pathway in sepsis

To date, treatment strategies for septic patients remain limited, relying mainly on antibiotic management and intensive care (170). The pathogenesis of sepsis is complex and multifaceted, affecting almost all bodily systems and organs, and exhibiting diverse pathophysiological presentations during each stage, posing challenges for clinical management (171). A series of specific therapies have been developed based on the pathogenesis of sepsis. Although they have shown significant efficacy in animal models of sepsis or *in vitro* experiments, none has been conclusively proven effective in clinical trials to date. In addition to the complexity of sepsis, clinical trials have certain inherent limitations, such as inconsistent inclusion and exclusion criteria among clinical trials and some indicators that do not adequately reflect clinical efficacy (172, 173). Current targeted drugs for sepsis focus on TLRs and neutralization of pro-inflammatory factors, but the clinical effects are not significant (174, 175). GSDMs proteins are co-ultimate executors of pyroptosis and inflammatory factors release, so GSDMs proteins are seen as novel and ideal targets for therapeutic agents of sepsis. A number of drugs have been shown to inhibit GSDMs-mediated pathways or GSDMs pore formation (**Table 3**), and these studies have also contributed to the development of novel effective drug targeted GSDMs proteins for the clinical treatment of sepsis.

**Table 3:** The therapy targeting GSDMs in sepsis.

Drug	Gasdermin	Study model	Detailed dosage	Disease	Mechanism	Ref (PMID)
Inhibition of GSDMs pathway						
Irisin	GSDMD	LPS or CLP induced mice LPS-induced H9c2 cells	1 μg/kg/day i.p. for three days 5 nM for 10 h	SCID	Attenuated sepsis-induced cardiac dysfunction by suppressing GSDMD-induced pyroptosis via the mitochondrial ubiquitin ligase-dependent mechanism.	35653888
Syringaresinol	GSDMD	CLP-induced mice LPS-induced H9c2 cells	50 mg/kg i.g. for 24 h 100 μmol/L for 6 h	SCID	Ameliorated SICD via the estrogen receptor/SIRT1/NLRP3/GSDMD pathway.	34801532
Honokiol	GSDMD	LPS-induced ALI rats LPS+ATP-induced BEAS-2B cells	5 mg/kg i.p. for 24 h after LPS 50 mM for 20 h	Sepsis induced-ALI	Alleviated LPS-induced ALI by inhibiting NLRP3 inflammasome-mediated pyroptosis via Nrf2 activation.	34844623
Tetramethylpyrazine	GSDMD	LPS-induced ALI mice RAW 264.7 and Ana-1 cells	50 mg/kg i.p. for 1 h before LPS 10 μg/ml for 24 h	Sepsis induced-ALI	Inhibited the TLR4/TRAF6/NF-κB /NLRP3/caspase-1 and TLR4/caspase-8 signaling pathways to reverse macrophages polarization and reduce cell pyroptosis	36416076
Metformin	GSDMD	LPS-induced ALI mice LPS-induced ECs	50 mg/kg i.p. for 0.5 h before LPS 10 mM for 24 h	Sepsis induced-ALI	Alleviated LPS-induced ALI by upregulating the expression of sirtuin 1 to inhibit NF-κB-NLRP3-mediated endothelial cells pyroptosis.	35910360
Fudosteine	GSDMD	CLP-induced septic mice	50 mg/kg i.g. for 1 h before CLP	Sepsis induced-ALI	Inhibited pyroptosis via the TXNIP/NLRP3/GSDMD pathway	35609679
Mangiferin	GSDMD	LPS-induced BMDMs	100 μg/mL for 24 h	Sepsis	Suppressed NF-κB/NLRP3/GSDMD Signaling Cascades to inhibited pyroptosis	36077522
Scutellarin	GSDMD	LPS-induced macrophages	50 μmol/L for 17 h	Sepsis	Inhibited activation of caspase-11 and NLRP3 via protein kinase A signaling to suppress GSDMD-mediated pyroptosis in vitro.	33532184
Xuebijing	GSDMD	CLP-induced mice	18 ml/kg i.v. for 24 h	Sepsis induced-ALI	Protected against septic ALI by reversing GSDMD-related pathway to inhibit NETs formation.	36467039
Ketone musk	GSDMD	LPS and ATP induced J774A.1 cells	40 μg/ml for 12 h	Sepsis	Inhibited the assembly of NLRP3 inflammasome and activation of caspase-1/GSDMD	30952097
Emodin	GSDMD	LPS-induced ALI rat and J774A.1 cells	80mg/kg i.g. for 0.5 h before LPS 80 μg/mL for 24 h	Sepsis induced-ALI	Suppressed caspase-1/GSDMD pathway by inhibiting NLRP3 to show protective effects	34787801
Emodin	GSDMD	LPS-induced 1321N1 cells	20 μM for 24 h	Sepsis brain injury	Inhibited the inflammation and pyroptosis of SBI by inactivating METTL3 -mediated NLRP3 expression in vitro.	35246004
Polygonatum sibiricum polysaccharides	GSDMD	LPS-induced SALI mice	300 mg/kg i.g. for 7 consecutive days	Septic acute liver injury	Inhibited NLRP3/caspase-1/GSDMD pathway-induced hepatocyte pyroptosis	36144734
Samotolisib	GSDMD	LPS-induced mice LPS-induced RAW264.7 cells	5 mg/kg for 5 days 10 μM for 24 h	Septic acute liver injury	Improved survival and reduced macrophage pyroptosis in septic mice through inhibiting caspase-11/GSDMD-mediated pyroptosis via regulating PI3K/AKT/mTOR/Nedd4 signaling	34483936
Baicalein	GSDMD	LPS+D-gal-induced mice LPS+D-gal-induced hepatocytes	30 mg/kg i.p. for 0.5 h before LPS/D-gal 10 mM for 12 h	Septic acute liver injury	Attenuated infection-mediated acute liver injury by blocking NLRP3/GSDMD-mediated pyroptosis	33272570
Inhibition of pore-forming GSDMs						
Disulfiram	GSDMD	LPS-induced septic in mice LPS-induced THP-1 and BMDM cells	Before LPS: 50 mg/kg i.p. for 24 h 50 μM for 2 h	Sepsis	Covalently modified human/mouse Cys191/Cys192 in GSDMD to block pore formation, thereby preventing IL-1β release and pyroptosis.	32367036
Tea polyphenols nanoparticles	GSDMD	LPS-induced mice LPS-induced RAW264.7 cells	25 mg/kg i.p. 3 times daily for 4 days 50 μg/mL for 24 h	Sepsis	Inhibited oligomerization of GSDMD and cell pyroptosis by scavenging RONS in endotoxin-induced sepsis.	35133795
NSA	GSDMD	LPS-induced septic mice and iBMDMs	20 mg/kg i.p. for 6 h 20 μM for 1 h	Sepsis	Suppressed the oligomerization of GSDMD-N dimer by directly binding to the Cys191 site of GSDMD, thus performing therapeutic effects in septic mice	30143556
PEITC	GSDMD	LPS-induced SALI and AML12 cells	30 mg/kg, i.g. for 3 consecutive days 3 µM for 1 h	Septic acute liver injury	Directly inhibited the Cys191 site of GSDMD to inhibit hepatocyte pyroptosis	35173734

H9c2, Rat cardiomyocytes; BEAS-2B, Human bronchial epithelioid cells; ECs, Endothelial cells; RONS, Reactive oxygen and nitrogen species; AR42J cell, Rat pancreatic exocrine cell; AML12, alpha mouse liver 12 cells.

### 4.1 Inhibition of GSDMs-mediated pathway activation

Melatonin reduced mortality in LPS-induced ALI mice and pyroptosis of human alveolar epithelial cells and macrophages by inhibiting the activation of NLRP3/caspase-1/GSDMD via Nrf2/heme oxygenase 1 pathway (176). In sepsis, damage of the endothelial barrier contributes to tissue ischemia and hypoxia, and one of the target organs is the heart. Irisin attenuated sepsis-induced cardiac dysfunction (SICD) by inhibiting GSDMD-induced pyroptosis through the mitochondrial ubiquitin ligase-dependent mechanism (177). Syringaresinol, a natural abstract, ameliorated SICD via the estrogen receptor/sirtuin 1/NLRP3/GSDMD pathway (178). The lungs are the most vulnerable organ in the progression of sepsis, and septic patients often present with ALI or ARDS at the early stage (179). Honokiol alleviated LPS-induced ALI by inhibiting NLRP3 inflammasome-mediated pyroptosis through Nrf2 activation (180). Tetramethylpyrazine, an effective compound extracted from the umbelliferous plant Chuanxiong, decreased infiltration of inflammatory cells and pro-inflammatory factors in the alveoli and reduced mortality in mice with LPS-induced ALI by inhibiting TLR4/TRAF6/NF-κB pathway to downregulate NLRP3 expression and caspase-1-mediated pyroptosis (181). Metformin alleviated LPS-induced ALI by upregulating the expression of sirtuin 1 to inhibit NF-κB-NLRP3-mediated endothelial cells pyroptosis (182). Fudosteine, a cysteine derivative, attenuated lung inflammation response and oxidative stress in septic mice via the TXNIP/NLRP3/GSDMD pathway (183). Mangiferin, a flavonoid widely distributed in several herbs, inhibited NLRP3/caspase-1/-11-mediated GSDMD activation in sepsis (184). Scutellarin, a flavonoid from Erigeron breviscapus, inhibited activation of caspase-11 and NLRP3 inflammasome via protein kinase A signaling to suppress GSDMD-mediated pyroptosis and the release of inflammatory mediators in LPS-induced macrophages (185). Xuebijing protected against septic ALI by inhibiting GSDMD-associated neutrophil extracellular traps formation (186). Ketone musk and Emodin, respective components of native musk and Chinese medicine Dahuang, enhanced cell viability and inhibited the release of pro-inflammatory cytokines by inhibiting the assembly of NLRP3 inflammasome and activation of caspase-1/GSDMD in LPS-induced macrophages. Emodin showed a significant protective effect against septic ALI (187, 188). In addition, emodin inhibited inflammation and pyroptosis of septic brain injury by inactivating m6A-mediated NLRP3 expression *in vitro (* 189). Liver is the major site of bacterial endotoxin-induced inflammation in sepsis. Polygonatum sibiricum polysaccharides significantly downregulated neutrophil infiltration and pro-inflammatory factor release in liver tissue to reduce 48-hour mortality in septic acute liver injury mice, primarily by inhibiting NLRP3/caspase-1/GSDMD pathway-induced hepatocyte pyroptosis (190). Samotolisib improved survival and reduced macrophage pyroptosis in septic mice by inhibiting caspase-11/GSDMD-mediated pyroptosis (191). Baicalein attenuated infection-mediated acute liver injury by blocking NLRP3/GSDMD-mediated pyroptosis (192).

### 4.2 Inhibition of pore formation

Disulfiram, a drug for the treatment of alcohol dependence, is an inhibitor of GSDMD pore formation. Disulfiram covalently modified GSDMD at Cys191/Cys192 (human/mouse) to block pore formation without affecting the cleavage of IL-1β and GSDMD, preventing IL-1β release and pyroptosis (193). Tea polyphenols nanoparticles scavenged reactive oxygen and nitrogen species via polyphenols-derived structure to inhibit oligomerization of GSDMD and cell pyroptosis in endotoxin-induced sepsis (194). Necrosulfonamide (NSA) inhibited pyroptosis and pro-inflammatory cytokines release in mice or human LPS and nigericin-induced monocytes/macrophages and reduced lethality in LPS-induced septic mice. NSA suppressed oligomerization of GSDMD-N dimer by directly binding to GSDMD at Cys191, thereby affecting GSDMD pore formation at the plasma membrane (195). Phenethyl isothiocyanate (PEITC), a natural compound found in cruciferous vegetables, may inhibit LPS-induced septic liver injury in a dose-dependent manner. PEITC could directly inhibit GSDMD at Cys191 and thus inhibit hepatocyte pyroptosis, which may provide a potential therapeutic strategy for septic liver injury (196).

## 5 Conclusion

Since the mortality of sepsis has been persistently high and is a major problem in critical care medicine, many researchers have conducted studies on molecular mechanisms and therapeutic targets in the pathophysiological process of sepsis. GSDMs proteins play important roles in the pathogenesis of sepsis and also aggravate the multi-organ dysfunction induced by sepsis. Among them, four classes are implicated in sepsis, including GSDMA, GSDMB, GSDMD and GSDME, with GSDMD being the most widely studied. More than one pathway may be operational at one time. Many molecules are also involved in the regulation of GSDMs-mediated pathways, such as epigenetic regulation and regulation of oxidative stress. All mechanisms may be involved in the different stages seen in sepsis. The sparking interests of GSDMs in sepsis is acting as pore-forming protein mediating cell pyroptosis or inflammatory factors release. The GSDMs pathway may be a promising therapeutic target for sepsis.

## Author contributions

WW conceived the study, data analysis, and drafted the manuscript. ZH conceived the study, its design and critically revised the manuscript. All authors read and approved the final manuscript.
